# Visual P2p component responds to perceived numerosity

**DOI:** 10.3389/fnhum.2022.1014703

**Published:** 2022-11-01

**Authors:** Paolo A. Grasso, Irene Petrizzo, Camilla Caponi, Giovanni Anobile, Roberto Arrighi

**Affiliations:** ^1^Department of Neuroscience, Psychology, Pharmacology and Child Health, University of Florence, Florence, Italy; ^2^Department of Physics and Astronomy, University of Florence, Florence, Italy

**Keywords:** EEG, event–related potentials (ERP), numerosity perception, adaptation, visual perception

## Abstract

Numerosity perception is a key ability for human and non-human species, probably mediated by dedicated brain mechanisms. Electrophysiological studies revealed the existence of both early and mid-latency components of the Electrophysiological (EEG) signal sensitive to numerosity changes. However, it is still unknown whether these components respond to physical or perceived variation in numerical attributes. We here tackled this point by recording electrophysiological signal while participants performed a numerosity adaptation task, a robust psychophysical method yielding changes in perceived numerosity judgments despite physical numerosity invariance. Behavioral measures confirmed that the test stimulus was consistently underestimated when presented after a high numerous adaptor while perceived as veridical when presented after a neutral adaptor. Congruently, EEG results revealed a potential at around 200 ms (P2p) which was reduced when the test stimulus was presented after the high numerous adaptor. This result was much prominent over the left posterior cluster of electrodes and correlated significantly with the amount of adaptation. No earlier modulations were retrievable when changes in numerosity were illusory while both early and mid-latency modulations occurred for physical changes. Taken together, our results reveal that mid-latency P2p mainly reflects perceived changes in numerical attributes, while earlier components are likely to be bounded to the physical characteristics of the stimuli. These results suggest that short-term plastic mechanisms induced by numerosity adaptation may involve a relatively late processing stage of the visual hierarchy likely engaging cortical areas beyond the primary visual cortex. Furthermore, these results also indicate mid-latency electrophysiological correlates as a signature of the internal representation of numerical information.

## Introduction

Both human and non-human species are endowed with a remarkable ability to make rapid and reasonably accurate estimates of the number of objects in a scene without the necessity to count (Dehaene, 2011). It is generally thought that this ability is mediated by a dedicated brain system capable of extracting numerosity information to guide behaviors (Nieder, 2016). This conceptualization has been supported by various fMRI studies revealing a robust signature of number representation in cortical regions located within the parietal cortex [see Feigenson et al. (2004) and Piazza and Izard (2009) for reviews]. For instance, Piazza et al. (2004) showed that the inferior parietal sulcus (IPS) responds to changes in numerosity with this pattern being evident also in preschoolers (Cantlon et al., 2006). On top of this, Harvey et al. described the existence of a topographically organized map for numbers in the human posterior parietal cortex with different populations of neurons tuned to different numerosities (Harvey et al., 2013; Harvey and Dumoulin, 2017).

Alongside, a series of studies exploited the high temporal resolution of electrophysiological (EEG) to investigate short latency characteristics of visual numerical processing. Results suggest the existence of a two-stage processing of numerical information. The first stage has been associated with an early negative ERP component peaking from about 75–150 ms post stimulus onset that is modulated by changes in cardinal numerosity (Hyde and Spelke, 2009; Park et al., 2016; Fornaciai and Park, 2017). The second stage has been identified in a positive posteriorly distributed ERP component peaking at around 200–250 ms (i.e., P2p). This latter component, originating from the parietal cortex, was first found to be modulated by the numerical distance between two values within relatively large numerical ranges (Libertus et al., 2007; Hyde and Spelke, 2009, 2012). More recently it has been shown that P2p scales according to the absolute numerosity of the stimulus rather than by its relative value (Park et al., 2016; Fornaciai and Park, 2017). In other words, P2p was not modulated by a comparison process between two quantities but represented the modulation of cortical signals elicited by the representation of different numerosity a result that, in turn, suggests that numerosity could be processed through a direct relationship between stimulus numerosity and the associated neural activity. Such conceptualization is indeed in line with the organization described in humans (Harvey et al., 2013; Harvey and Dumoulin, 2017; Cai et al., 2021) and also with a single neuron recording primate study finding a monotonic modulation of neural response to numerosity in monkey lateral intraparietal cortex (Roitman et al., 2007; Nieder, 2016).

Although evidence described above suggests a functional mechanism capable of extracting the numerical value of visual stimuli, it is still unclear whether this network encodes perceived or physical numerosity. Previous studies have shown that the perception of the numerosity is robustly distorted in several conditions such as when a subset of the stimuli in a set get connected (He et al., 2009; Pomè et al., 2021), grouped by spatial proximity or similarity of a salient feature (Zhao and Yu, 2016; Yu et al., 2019) or presented in a region of space where relatively higher or lower numerous stimuli had been previously displayed (Burr and Ross, 2008; Arrighi et al., 2014). In other words, much evidence suggests that numerosity processing might occur along the hierarchy of visual areas with a relatively noisy mapping of physical quantity. However, at present, literature missed to identify whether neural correlates of numerical processing mirror physical or perceived changes as most of the studies employed either passive exposure to numerical stimuli or active paradigms that did not allow to distinguish between the two components. To this aim, we here combined EEG recording with psychophysical numerosity adaptation, a phenomenon causing a robust underestimation of the perceived numerosity of the items in a set as a consequence of a prolonged exposure to a relatively highly numerous array of dots (Burr and Ross, 2008). The prediction is clear, if event-related potential (ERP) components associated with numerical processing follow changes in physical numerosity then they should be unaffected by adaptation and should scale only in case of real numerical variations. Conversely, if they reflect perceived numerosity changes, a modulation along with the magnitude of adaptation should be expected.

## Materials and methods

### Participants

Sample size was determined through a power analysis conducted using G*Power 3 Software (Faul et al., 2007). The analysis indicated that a total sample of 25 participants would be needed to detect medium effects (f = 0.30) with 90% power and an alpha level of 0.05. Twenty-eight healthy participants took part in the study. Three participants were excluded because of ERP response being extremely corrupted by alpha activity. The final sample then comprised twenty-five participants (mean age: 25.16 years, SD: 5.03 years; eight males, one author). All participants were right-handed, had normal or corrected to normal visual acuity and provided written informed consent.

The research was approved by the local ethics committee (“*Commissione per l’Etica della Ricerca,”* University of Florence, 23rd September 2021, n. 174).

### Apparatus and stimuli

The whole experiment was conducted in a dimly lit and sound attenuated room with participants seated 51 cm away from an LED monitor (20 inches, refresh rate: 60 Hz, 1,920 × 1,080-pixel resolution) with their head placed on a chinrest.

Stimuli comprised of clouds of white non-overlapping random dots drawn within a virtual 11° diameter circle with center at 11 left or right of a central fixation cross and presented on a gray background. Total surface area (i.e., cumulative area covered by dots) was kept constant across all stimuli by scaling the diameter of the dots: Similarly, convex hull remained fixed across all stimuli employed. These manipulations allowed to carefully control for brightness levels across stimuli.

### Experimental paradigm and procedure

**FIGURE 1:**
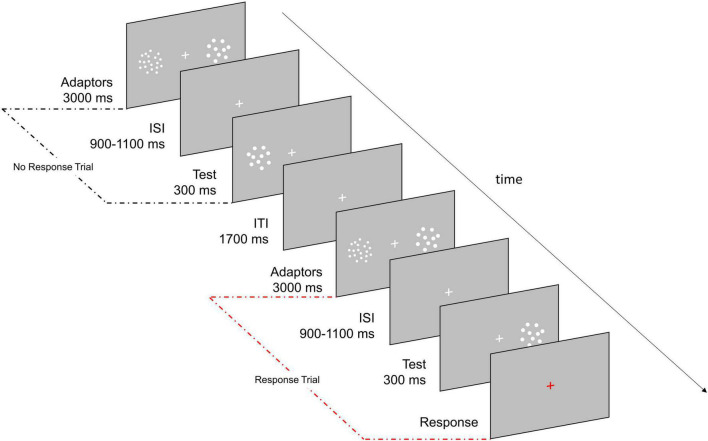
Experimental paradigm. Participants were asked to estimate the numerosity of the test stimulus (22, 26, 30, 35, or 41 dots) and to verbally report it only if the fixation cross turned red (33% of trials). During adaptation the test could appear either in the location previously occupied by the High Adaptor (a cloud of 48 dots; Adaptation condition) or by the Neutral Adaptor (a cloud of dots matching the numerosity of the test; Neutral condition). In half of the blocks the High Adaptor was presented on the **left** while in the other half it was presented on the **right**. Baseline consisted in trials in which the test stimuli were not preceded by any adaptation period.

Each trial began with a 3,000 ms adaptation period during which two clouds of dots (i.e., adaptors) concurrently appeared on the left and right side of the fixation cross. One of the two clouds always contained 48 dots (i.e., High Adaptor) while the other one (i.e., Neutral Adaptor) matched the numerosity of the following test stimulus and was thus not expected to induce any perceptual distortion in numerosity estimates (Burr and Ross, 2008; Aagten-Murphy and Burr, 2016; Fornaciai et al., 2016; Grasso et al., 2021a,b). The adaptation period was followed by an ISI (randomly jittered between 900 and 1,100 ms) which preceded the presentation of the test stimulus (300 ms), a cloud of 22, 26, 30, 35, or 41 dots randomly appearing in the location of the High or Neutral Adaptor (Figure 1). Participants were asked to mentally estimate the numerosity of the test stimulus and to verbally report it only when the fixation cross turned red (33% of trials). This procedure ensured participants kept focused on the task throughout the session while allowed a shortened experiment duration. Baseline consisted in simple estimation trials not being preceded by any adaptation period. Each numerosity was presented 48 times during adaptation trials (24 times in the location of the High Adaptor and 24 times in the location of the Neutral Adaptor) and 24 times during baseline trials except for the central numerosity (i.e., 30) which was presented 480 times during adaptation (240 times in the location of the High Adaptor and 240 times in the location of the Neutral Adaptor) and 240 times during baseline. The larger amount of trials allocated to the central numerosity was exploited to boost sensitivity at unveiling the neural correlates of perceived numerical changes related to adaptation induced distortions. All remaining numerosities (i.e., 22, 26, 35, and 41) mostly served as filling trials and were instead exploited to map neural correlates mirroring changes in physical numerosity.

Adaptation trials were divided in ten blocks, half of which had the High Adaptor on the left side and the Neutral Adaptor on the right side, and the other half with the opposite adaptors’ arrangement. When the High Adaptor was on the left (Neutral Adaptor on the right), the Adaptation condition corresponded to test presented on the left while the Neutral condition corresponded to test presented on the right and vice versa when the High Adaptor was on the right. This allowed to have half of the trials presented on the left and half on the right for both Adaptation and Neutral conditions to discard potential differences due to the location of the stimuli. Similarly, baseline trials were presented throughout the five blocks with stimuli appearing half on the left and half on the right hemifield.

The order of Baseline and Adaptation blocks was pseudo randomized. To get familiarized with the numerosity range, before the experiment, participants performed a few training trials in which feedback about the target numerosity was provided.

### Electrophysiological recording and preprocessing

Electrophysiological signal was recorded with a g.Nautilus Multi-Purpose system (gTEC, Schiedlberg, Austria) from 30 g.SCARABEO active gel-based electrodes (FP1, FP2, F7, F3, Fz, F4, F8, FC5, FC1, FC2, FC6, C3, Cz, C4, CP5, CP1, CP2, CP6, P7, P3, Pz, P4, P8, PO7, PO3, POz, PO4, PO8, O1, O2) while electrooculogram (EOG) signal was recorded from two electrodes positioned on the outer canthi of both eyes. The signal was referenced online to the right earlobe and the ground electrode was placed on AFz. Impedances were kept below 30 kΩ. The signal was recorded with a high-pass filter of 0.01 Hz and digitized at a sampling rate of 500 Hz. Due to technical issues, a very small percentage of triggers were not successfully delivered (average missed trigger signals–Baseline: 0.3%; Adaptation: 0.5%) and the corresponding trials were thus removed also from behavioral data.

Pre-processing was carried out using custom routines in MATLAB R2020b (The MathWorks, Inc., Natick, MA, USA) and EEGLAB v2020.0 (Delorme and Makeig, 2004). First, the signal was downsampled to 250 Hz and band-pass filtered from 1 to 40 Hz (type: FIR; cut-off frequency: –6 dB; 0.5 40.5 Hz). Subsequently, bad channels were interpolated (average interpolated channels–Baseline: 1.2; Adaptation: 1.5) and signal was offline re-referenced to the average of all electrodes. Epochs (–500–1,000 ms) corresponding to the presentation of the central numerosity (i.e., 30) were extracted from the continuous EEG and those containing muscular artifacts or blinks during stimulus presentation were discarded by visual inspection (average removed epochs–Baseline: 5.7%; Adaptation: 4%). Infomax independent component analysis (ICA) algorithm was run and components corresponding to eye-movements and to residual anterior muscle artifacts were removed (average removed ICs–Baseline: 4.9; Adaptation: 4.9). Finally, epochs were trimmed from –200 to 600 ms and baseline period was removed (–200–0).

To measure ERP responses elicited by physical numerosity changes we also analysed the electrophysiological signal produced by the presentation of filling trials (i.e., trials in which the test stimulus was composed of 22, 26, 35, or 41 dots). To increase the amount of trials per numerosity, we merged Baseline and Neutral conditions after controlling that numerical estimates obtained in the two conditions closely matched (see Section “Behavioral” in “Results” below for further details). More specifically, epochs (–500–1,000 ms) collected at Baseline and in the Neutral conditions during the presentation of numerosities 22, 26, 35, and 41 were selected. This led to obtain a total of 48 trials for each numerosity. We followed the same pre-processing steps used for the central numerosity that is, we first discarded epochs containing muscular artifacts or blinks during stimulus presentation (average removed epochs: 5.1%) and then removed components corresponding to eye-movements and residual anterior muscle artifacts (average removed ICs: 5.2). Finally, epochs were trimmed from –200 to 600 ms and baseline was removed (–200–0).

## Results

### Behavioral

**FIGURE 2:**
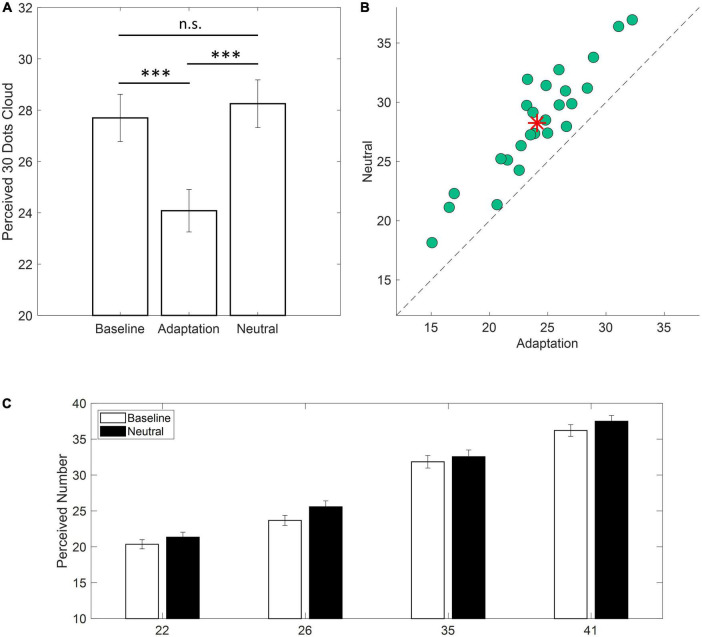
Behavioral results. **(A)** Average estimates of a cloud of 30 dots in the three experimental conditions (i.e., Baseline, Adaptation, Neutral). Error bars are ± 1 SEM (***p < 0.001). **(B)** Scatterplot depicting single participants responses in the Adaptation condition against the Neutral condition. Data falling above the equality line indicate lower estimation values for the Adaptation condition. Red asterisk indicates the average across participants. **(C)** Average estimates for clouds containing 22, 26, 35, or 41 dots for the Baseline and Neutral conditions (white and black bars, respectively). Error bars are ± 1 SEM.

At first, we selected the responses to the central numerosity (i.e., 30) in the three experimental conditions (i.e., Baseline, Adaptation, Neutral). For each participant and each experimental condition, numerosity estimates lying above or below twice the upper or lower quartile of the response distribution were discarded. The number of trials eliminated *via* this pruning procedure, mainly exploited to discard errors in response dialing, was very low and corresponded to an average of 1.16% in Baseline, 2.08% in the Adaptation condition and 0.41% in the Neutral condition. In light of the typical adaptation aftereffects (Grasso et al., 2021a,b) we expected roughly accurate numerosity estimates in the Baseline and the Neutral conditions. Conversely, a consistent numerosity underestimation was to be expected in the Adaptation condition. As evident from Figure 2A, the expectations were confirmed. In the Baseline and Neutral conditions, estimates were similar and, on average, very close to the veridical value (i.e., Baseline: 27.7; Neutral: 28.2). On the other hand, in the Adaptation condition, numerosity was consistently underestimated as the test stimulus was perceived as containing about 24 dots (∼20% underestimation). This result was confirmed by the one-way ANOVA with the within factor Condition (Baseline, Adaptation, Neutral) which revealed a highly significant main effect [*F*_(2_,_48)_ = 128.21; *p* < 0.001; *η^2^* = 0.646, LogBF_10_ = 19.6] explained by Adaptation being different from both Baseline and Neutral (all *p*s < 0.001; Tukey-Kramer correction) and Baseline not being different from Neutral (*p* = 0.49). Importantly, all participants exhibited an underestimation of the test stimulus in the Adaptation condition as compared to the Neutral condition (Figure 2B) confirming a consistent adaptation effect.

To test whether numerical estimates obtained during Baseline and Neutral conditions did not significantly differ, a two-ways ANOVA was conducted on numerical estimates of filling trials (i.e., 22, 26, 35, and 41). The main effect of Condition was not significant [*F*_(1,24)_ = 3.619; *p* = 0.07; *η^2^* = 0.008] and the same held true for the interaction Condition x Numerosity [*F*_(3,72)_ = 0.826; *p* = 0.433; *η^2^* = 0.001] confirming that the Neutral Adaptor did not produce any bias in the estimation of numerical quantities which closely matched those reported at Baseline (Figure 2C).

### Event related potentials

A large cluster of posterior electrodes was *a priori* chosen based on previous literature reporting numerical processing being associated with changes in ERP responses within occipito-parietal sensors (Hyde and Spelke, 2009, 2012; Park et al., 2016; Fornaciai and Park, 2017). ERP was derived from the average response recorded over P3, P4, P7, P8, PO3, PO4, PO7, PO8, O1, and O2 electrodes. For statistical analysis we applied a robust non-parametric approach allowing to detect differences between the ERPs in the three experimental conditions (i.e., Baseline, Adaptation, Neutral) at all-time points without focusing *a priori* on any specific components (e.g., P1, N1, P2). In details, for each time point from stimulus onset until the end of the epoch, a bootstrap distribution (20,000 iterations) of the difference between paired conditions (i.e., Baseline vs. Adaptation; Baseline vs. Neutral; Adaptation vs. Neutral) was built and the probability to reject the null hypothesis (i.e., no difference) was computed at each time point. False discovery rate correction was applied, and significant values were considered reliable only if evident across a minimum of four consecutive time points (i.e., 16 ms). It is to be noted that the use of a Neutral Adaptor matching the High Adaptor in total surface area and convex hull allowed to distinguish between the effects purely related to numerosity adaptation and those mediated by the prolonged exposure to a bright stimulus not biasing subsequent numerosity estimates. Following this rationale, a change in ERP response was expected to reflect perceptual changes in numerosity perception only when spotting differences between the Adaptation and the Neutral conditions. Conversely, a change in ERP response, evident both in the Adaptation and in the Neutral conditions would be considered to reflect an artifact of the prolonged exposure to the bright pattern of the adaptor or other processes not strictly related to changes in numerosity processing.

**FIGURE 3:**
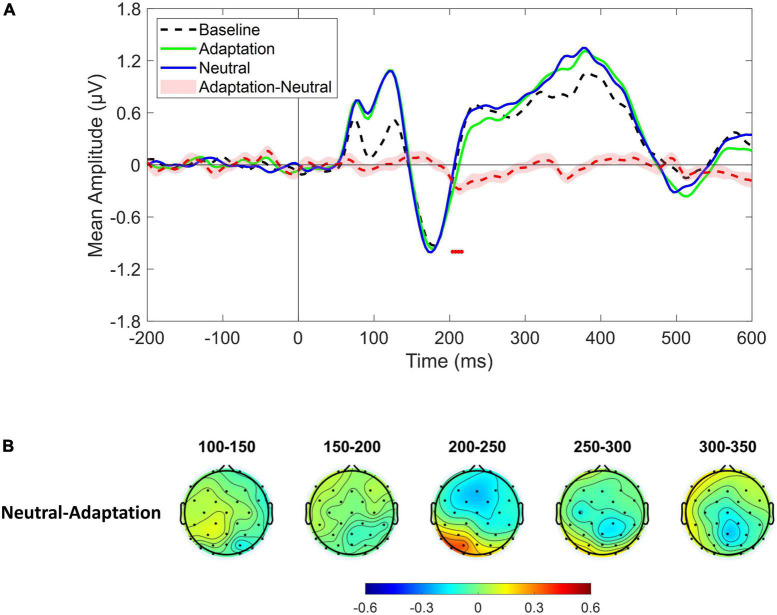
**(A)** Event-related potentials (ERPs) response averaged across electrodes P3, P4, P7, P8, PO3, PO4, PO7, PO8, O1, O2 for Baseline (dotted black ERP), Adaptation (green solid ERP), and Neutral (blue solid ERP) conditions. Red dotted curve depicts the temporal distribution of the difference (plus standard error) between Adaptation and Neutral ERP response while red dots depict the time interval of statistical significance. **(B)** Scalp topographies depicting the spatial and temporal distribution of the difference between Neutral and Adaptation conditions.

Event–related potentials derived from the whole cluster of electrodes revealed a significant amplitude increase in the time range of the P1 component (∼80–140 ms) that was evident both in the Adaptation and in the Neutral conditions. More interestingly, the Adaptation condition showed a significant reduction with respect to the Neutral condition in the time range of the P2p component (∼200–220 ms) as depicted by red dots in Figure 3A. Furthermore, the spatial distribution of the difference revealed that the larger variation was evident over left posterior sensors suggesting a partial lateralization of such an effect (Figure 3B).

**FIGURE 4:**
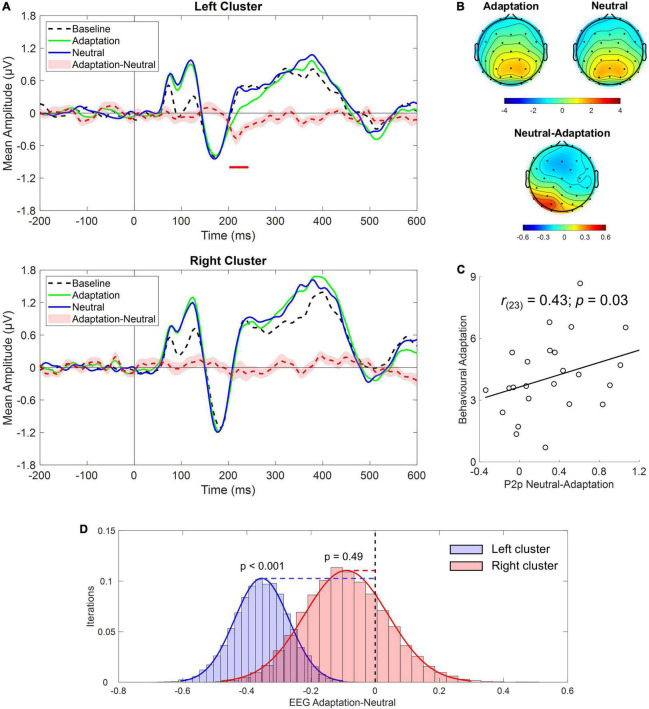
**(A)** Event-related potentials (ERPs) response averaged across electrodes P3, P7, PO3, PO7, O1 **(upper panel)** and P4, P8, PO4, PO8, O2 **(bottom panel)** during Baseline (dotted black), Adaptation (green solid), and Neutral (blue solid) conditions. Red dotted curve depicts the temporal distribution of the difference (plus standard errors, light red shading) between Adaptation and Neutral conditions while red dots depict the time interval of statistical significance. **(B)** Scalp topographies depicting the spatial distribution within the time range of significance for the Adaptation, Neutral and the difference between the two. **(C)** Correlation between behavioral adaptation magnitude and electrophysiological response difference between the Adaptation and the Neutral conditions obtained in the left cluster of electrodes. **(D)** Bootstrap distributions (20,000 iterations) of the EEG difference between Adaptation and Neutral conditions within the time range of the P2p component for the left (blue histogram) and the right cluster (red histogram) of electrodes. The null hypothesis (i.e., no difference between Adaptation and Neutral conditions), indicated by the black vertical dotted line, falls outside the distribution for the left but not for the right cluster.

Following this clue and in light of previous reports of cortical lateralization of numerical processing (e.g., Chochon et al., 1999; Piazza et al., 2006; Pinel and Dehaene, 2010), the same analysis was conducted after selecting either the left (i.e., P3, P7, PO3, PO7, O1) or the right (i.e., P4, P8, PO4, PO8, O2) cluster of electrodes. Results revealed a significant difference in the P2p component between Adaptation and Neutral conditions which was evident only in the left cluster within the time range depicted by red dots in Figure 4A (∼200–250 ms; see also topographies on Figure 4B). To confirm that the P2p amplitude reduction retrieved in the left cluster was related to adaptation-mediated changes in numerosity perception, we correlated behavioral and electrophysiological response differences between Adaptation and Neutral conditions. The analysis revealed a positive significant correlation [Spearman; *r*_(23)_ = 0.43; *p* = 0.03] indicating that the larger was the behavioral adaptation effect, the larger was the difference in the associated P2p amplitude (Figure 4C). No difference between Adaptation and Neutral conditions was evident when the right cluster was considered (*p* = 0.49; Figure 4D). Furthermore, Adaptation and Neutral conditions produced a significant increase in the time range of the P1 component in both clusters while, for the right cluster, also an increase in the time range of the P3 component was evident.

**FIGURE 5:**
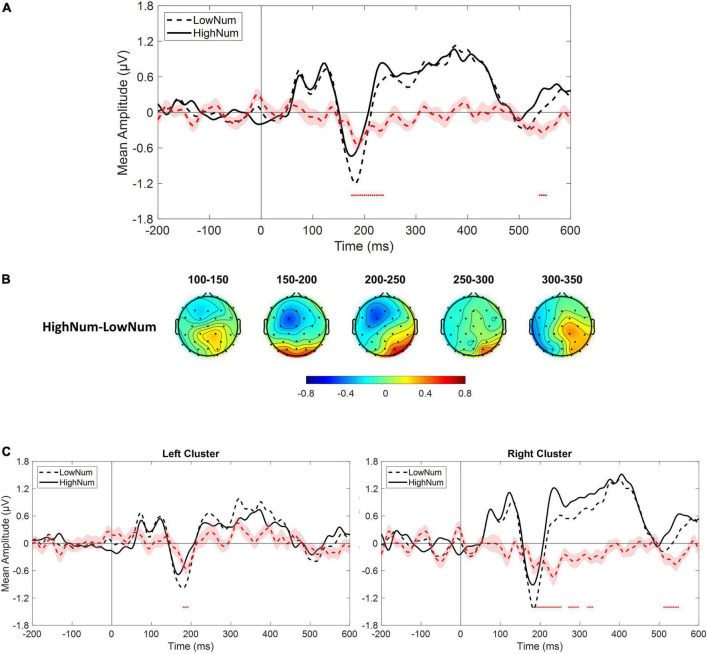
**(A)** Event-related potentials (ERPs) response averaged across electrodes P3, P4, P7, P8, PO3, PO4, PO7, PO8, O1, O2 for the presentation of lower numerical stimuli (LowNum condition) and higher numerical stimuli (HighNum condition). **(B)** Scalp topographies depicting the spatial and temporal distribution of the difference between HighNum and LowNum conditions. **(C)** ERPs response averaged across electrodes P3, P7, PO3, PO7, O1 **(left panel)** and P4, P8, PO4, PO8, O2 **(right panel)** during LowNum (dotted black) and HighNum (solid black) conditions. Red dotted curve always depicts the temporal distribution of the difference (plus standard errors, light red shading) between HighNum and LowNum conditions while red dots depict the time interval of statistical significance.

As a sanity check, we tested whether we could replicate previous results describing changes in both early and mid-latency electrophysiological components as a function of numerosity. Given the relatively low number of trials collected for numerosities different from the central one (i.e., 30), we collapsed trials from lower numerical values (i.e., 22 and 26; LowNum condition) and trials from higher numerical values (i.e., 35 and 41; HighNum condition) and compared the electrophysiological responses obtained in the two conditions by using the same non-parametric approach described above. Based on previous reports, we expected to find a reduced electrophysiological response as the number of elements decreased. This is exactly what we found as the LowNum condition produced a significantly lowered response as compared to the HighNum condition. This difference spanned from around 180–240 ms which is consistent with the time range of N1 and P2p components (Figure 5A). Interestingly, topographies (Figure 5B) revealed that this difference was bilaterally distributed in the time range of the N1 (i.e., 150–200 ms) while clearly lateralized toward right posterior sensors in the time range of the P2p (i.e., 200–250 ms). For this reason, the same analysis was again conducted after selecting either the left (i.e., P3, P7, PO3, PO7, O1) or the right (i.e., P4, P8, PO4, PO8, O2) cluster of electrodes. Results confirmed that while N1 was modulated in both clusters, P2p changed its amplitude only when the right cluster of electrodes was considered (Figure 5C).

In summary, on the one hand, our results confirm that changes in physical numerosity elicit a modulation in both early and mid-latency components of the electrophysiological response while, on the other hand, reveal that changes in perceived (but not physical) numerosity elicit a selective modulation of mid-latency P2p component with earlier components left unaffected. Interestingly, this pattern of results is also accompanied by an opposed topographical activation within the time range of P2p with right posterior sensors spotting differences in physical numerosity changes while left posterior sensors being sensitive to perceived changes.

## Discussion

In the current study we found that a component of the ERP response classically associated with numerical cognition (P2p) is modulated not only by physical changes but also by perceived variations in numerosity. We exploited an adaptation paradigm as a tool to dissociate perceived from physical changes in numerosity while electrophysiological signal was recorded. During the task participants were asked to estimate the numerosity of items in a set (i.e., test stimulus) consisting of a briefly displayed cloud of dots presented either in the location where a highly numerous dots array previously appeared (i.e., High Adaptor) or in the location where a cloud of dots with the same numerosity of the test stimulus was presented (i.e., Neutral Adaptor). According to previous studies, the latter condition was not expected to yield any bias in numerical estimates while the former was expected to produce a consistent underestimation of the perceived numerosity of the test (Burr and Ross, 2008; Aagten-Murphy and Burr, 2016; Fornaciai et al., 2016; Grasso et al., 2021a,b). Behavioral data confirmed this expectation revealing that all participants underestimated the numerosity of the test stimulus in the Adaptation condition (i.e., High Adaptor) with adaptation inducing a change in perceived numerosity of about 20%. On the contrary, numerosity estimates in the Neutral condition (i.e., Neutral Adaptor) were almost identical to those obtained in the Baseline condition, where no adaptation occurred. Importantly, by displaying a Neutral Adaptor matching the High Adaptor in terms of brightness, we could disentangle between the effects induced by the mere prolonged exposure to a bright pattern from those related to adaptation-mediated changes in numerical processing.

Electrophysiological results revealed a significant amplitude reduction in the time range of the P2p component (∼200–250 ms) that was specific for the Adaptation condition. Conversely, P2p in the Neutral condition was very similar to Baseline mirroring behavioral results. Interestingly, P2p reduction for the Adaptation condition was selectively evident within the left posterior cluster of electrodes while it was absent within the right cluster suggesting that the neural changes produced by numerosity adaptation could mainly entail a change in the activity of the left hemisphere. Furthermore, the reduced P2p amplitude in the left cluster correlated with the amount of adaptation-dependent underestimation of the test stimulus suggesting that the two phenomena might be related to a shared mechanism. Importantly, no evidence of earlier components being modulated by perceived numerical changes were retrieved. Specifically, the N1 components elicited by test stimulus being presented during Baseline, Adaptation or Neutral conditions were virtually overlapped. Similarly, the amplitude modulation within the time range of the P1 component (∼100 ms) was found in both Adaptation and Neutral conditions suggesting that this variation was mostly unrelated to changes in perceived numerical estimates.

To note, when looking at ERP response elicited by physical numerosity changes we confirmed results reported by previous works in which both an early and a mid-latency modulation was found to occur (e.g., Park et al., 2016; Fornaciai and Park, 2017). We also showed that while early amplitude changes (i.e., N1) were retrievable bilaterally, this was not the case for P2p which was found to modulate its amplitude across right posterior sensors. Crucially, this topographical pattern is opposed to that retrieved for perceived numerical changes where left posterior sensors were found to be involved. This difference could potentially highlight the recruitment of segregate cortical regions reflecting different nuances of numerosity perception although we acknowledge that this interpretation goes beyond our data.

Numerosity coding is thought to result from a multistage process transforming sensory inputs into an abstract representation. In particular, earlier-latency components have been associated with a location-specific and attentional-dependent processing that would be crucial for the elaboration of very low numerical ranges (Hyde and Spelke, 2009; Wurm et al., 2021) while mid-latency components (i.e., P2p) would be involved in the processing of abstract location-invariant numerical information mostly evident within relatively large numerical ranges (Temple and Posner, 1998; Libertus et al., 2007; Hyde and Spelke, 2009; Park et al., 2016; Fornaciai and Park, 2017). Evidence of such multistage coding has been reported in previous EEG studies. For instance, in a recent paper Park and colleagues showed that both P2p and an earlier component peaking at around 75 ms scale with the number of stimuli presented. The larger was the number of dots the higher was the amplitude. The study carefully controlled for the influence of other non-numerical factors (e.g., total surface area, individual dots area, density) covarying with the increase in numerosity and found evidence of stronger sensitivity to numerical changes as compared to changes in other visual attributes (Park et al., 2016). A similar result was found by Fornaciai and Park within higher numerical ranges (i.e., texture-density regime) revealing a systematic modulation of the P2p component together with an earlier negative component peaking at around 100 ms (Fornaciai and Park, 2017).

Our results extend these findings by showing that P2p component reflects changes in perceived numerosity while the earlier components could be much more grounded to the physical characteristics of the stimuli. It is important to point out that we here employed a series of control procedures allowing to isolate variation in ERP response that are to be attributable to changes in the processing of numerical information. First, we always compared the ERP response under a condition of high adaptation vs. a condition of neutral adaptation. This allowed to spot ERP changes resulting from the prolonged presentation of a bright pattern rather than reflecting changes in numerical processing. Second, comparing ERP responses derived from the presentation of the same numerosity (i.e., 30) allowed us to exclude the influence of non-numerical attributes of the stimuli covarying with numerical processing. Finally, stimuli were always presented both on the left and on the right visual field to discard potential differences related to the absolute location in space. Controlling for these variables allowed us to select only those variation in the EEG signal that are purely related to perceived changes in numerosity encoding. The variation was selectively spotted within the time range of the P2p and correlated significantly with the behavioral change suggesting a tight link between the two measures. No significant differences between Adaptation and Neutral conditions were evident within earlier components. The only earlier variation in ERP response was spotted in the time range of the P1 component but this variation was retrievable both in the Adaptation and in the Neutral condition, suggesting that it had nothing to do with adaptation-driven perceptual changes and could be mostly related to the prolonged exposure to the bright pattern of the adaptors. Importantly, we also confirmed that both N1 and P2p are sensitive to variation in physical numerosity. However, when the variation is illusory induced by adaptation, N1 remains unchanged and only P2p component varies its amplitude.

It could be argued that what we here described is a variation in ERP responses related to high spatial frequencies adaptation rather than pure numerosity. Although potentially valid, as the spatial frequency of the adaptor was *de facto* higher than the spatial frequencies employed for the test, we consider it unlikely for several reasons. On the one hand, behavioral studies demonstrated that the encoding of numerosity information is possible even in case the spatial frequency was kept constant to make it uninformative to solve the task (Adriano et al., 2021, 2022) providing evidence for an independence of numerosity encoding to spatial frequency analysis. This result compliments the well-known results about the connectedness effect (He et al., 2009) in which the addition of lines to connect the dots in a display, despite increasing the pattern of high spatial frequency (as more sharp edges are displayed), provides a significant reduction of perceived numerosity to disentangle the two components. On the other hand, it is well-documented in both animal (e.g., Foster et al., 1985; Victor et al., 1994; Issa et al., 2000) and human studies (e.g., Mazer et al., 2002; Henriksson et al., 2008; Hallum et al., 2011) that the analysis of spatial frequencies is mostly subserved by early cortical areas such as V1. However, no evidence exists for the involvement of early visual cortices in numerosity adaptation but there exists for the lack of such an involvement. This is reported by recent fMRI evidence showing reliable post-adaptation decoding accuracy for IPS but not for V1 (Castaldi et al., 2016) but also by psychophysical evidence revealing a robust influence of visuospatial attentional mechanisms in building up numerosity adaptation that do not generalize to other adaptation tasks involving the activity of earlier visual areas (Grasso et al., 2021a,b). Furthermore, also our results suggest that numerosity adaptation likely affects cortical areas located within a relatively high hierarchical node while leaves activity within earlier cortical regions mostly unchanged. This is because while early component of the visual ERP response have been mostly associated with activity arising from striate or early extrastriate regions (Di Russo et al., 2002), the P2p component was found to originate from the parietal cortex (Hyde and Spelke, 2012; Fornaciai et al., 2017) and its amplitude was found to vary when low electrical current are applied over posterior parietal cortex (Grasso et al., 2020b,2021c). Finally, we recently showed that numerosity adaptation is selective to salient features, such as color, suggesting that adaptation to numerical quantities likely occurs after feature-binding and other processing contributing to object recognition (Grasso et al., 2022).

To conclude, the present results not only highlight the extraordinary ability of the visual brain to rapidly shape its responses based on varying external needs [for a recent review on visual plasticity see Grasso et al. (2020a)] but also that such neural response changes are selectively evident within the associated level of visual information coding. We found that only the P2p component is modulated by perceptual adaptation supporting the notion that late electrophysiological correlates of numerical processing do not reflect the actual physical number of elements in a scene but rather express a reworking of the numerical information that is closely bounded with subjective experience. These results suggest that the two identified stages of numerical coding could reflect sharply distinct processes: the first mostly linked to the analysis of physical characteristics of the stimuli and the second being much more bounded to higher-level information coding.

## Data availability statement

The raw behavioral and electrophysiological data have been deposited at Zenodo repository and are publicly available at the following address: https://doi.org/10.5281/zenodo.7186299.

## Ethics statement

The studies involving human participants were reviewed and approved by Commissione per l’Etica della Ricerca, University of Florence. The patients/participants provided their written informed consent to participate in this study.

## Author contributions

PG contributed to conception and design of the study, performed the statistical analysis, and wrote the first draft of the manuscript. IP and CC collected the data. RA and GA supervised the project. All authors contributed to manuscript revision, read, and approved the submitted version.
